# Protocol for Projecting Allele Frequency Change under Future Climate Change at Adaptive-Associated Loci

**DOI:** 10.1016/j.xpro.2020.100061

**Published:** 2020-07-15

**Authors:** Meghan Blumstein, Andrew Richardson, David Weston, Jin Zhang, Wellington Muchero, Robin Hopkins

**Affiliations:** 1 Department of Organismic and Evolutionary Biology, Harvard University, 26 Oxford Street, Cambridge, MA 02138, USA; 2 Center for Ecosystem Science and Society, Northern Arizona University, Flagstaff, AZ 86011, USA; 3 School of Informatics, Computing, and Cyber Systems, Northern Arizona University, Flagstaff, AZ 86011, USA; 4 Biosciences Division, Oak Ridge National Laboratory, Oak Ridge, TN 37831, USA; 5 The Arnold Arboretum, 1300 Centre Street, Boston, MA 02130, USA

## Abstract

We describe how to predict population-level allele frequency change at loci associated with locally adapted traits under future climate conditions. Our method can identify populations that are at higher risk of local extinction and those that might be prime targets for conservation intervention. We draw on previously developed community ecology statistical methods and apply them in novel ways to plant genomes. While a powerful diagnostic tool, our method requires a wealth of genomic data for use.

For complete details on the use and execution of this protocol, please refer to Blumstein et al. (2020).

## Highlights

A method for projecting adaptive allele frequency shifts under climate change
Can be used to identify vulnerable populations and help choose conservation targets
However, the method has large genomic and climate data requirements

## Graphical Abstract

**Figure:**
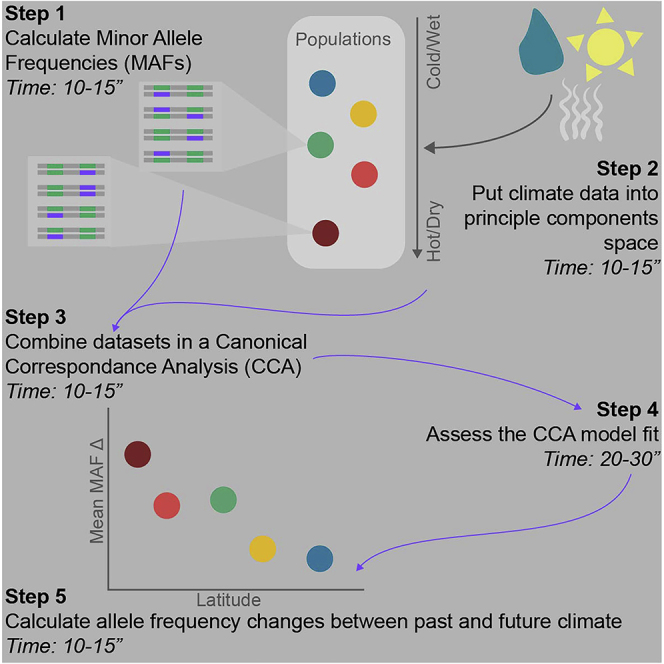

## Before You Begin

### Gather Data Resources

***Note:*** Example data can be found at https://github.com/blumsteinm/Projecting_MAF_ClimateChange/tree/master/Example_Data

1. Climate Data
a. Past Climate Normal information for population locations (.csv file format)
i. Normals data are three-decade averages of climate variables and are produced every 10 years. They are essential to use instead of daily or annual climate data as we are interested in questions of local adaptation to climate, not weather.
ii. We used 1 km resolution climate monthly Normals data from 1961–1990 from Climate WNA (http://www.climatewna.com/) (Hamann et al., 2013, Blumstein et al., 2020, Wang et al., 2012). These data were interpolated from the original 4x4 km WNA data using demography information, then point values were extracted using each genotype’s latitude and longitude of origin.
iii. Example Dataset is Climate_Normals_1961_1990.csv
b. Projected Climate Change information for population locations (.csv file format)
i. Climate projections should come from a Global Climate Model (GCM) or ensemble of several GCMs.

**CRITICAL:** ensure that the projected climate variable names match the normals climate variables for use in the statistical model in step 2 and all geographic data are in the same projection.

ii. We used 1 km resolution ensemble projections for the 2080 decade from ClimateWNA (Hamann et al., 2013, Blumstein et al., 2020, Wang et al., 2012).
iii. Example Dataset is Climate_Projections_Ensemble2080s.csv
2. Sequence Samples (.bed file format)
a. You will need genomic sequences for the genotypes in your study. These can be full genome sequences or subsets of sequences gathered by reduced sampling representation techniques (eg. RADSeq)
b. We used genomes resequenced by the Joint Genome Institute and made publicly available (Joint Genome Institute, D. O. E, 2019) for the genotypes in our study
c. All loci with MAF <3% should be excluded from analysis.
d. Example Dataset is POTR_SNPs_Subset [.fam/.bed/.bim]
3. Loci of Interest
a. A list of the genes or loci of interest in your study. These should be loci that contribute to or are associated with local adaptation. They can be pulled from the literature, previous work, or Quantitative Trait Loci (QTL)/Genome Wide Association Sudies (GWAS). Ideally these loci will show signatures of local adaptation in your own populations (Q_st_ > F_st_). If performing QTL or GWAS studies, it is important to correct for genetic relatedness/population structure in your model. See “Resources Availability” section for data resources such as *Phytozome* for plant genomic and genetic information.
b. We measured an adaptive trait in common gardens (Nonstructural Carbohydrate Storage (NSC)) and used this data to perform a Genome Wide Association Study (GWAS) with our genomic resequenced data, control for genetic relatedness in the model (Blumstein et al., 2020). This analysis identified loci that were significantly associated with our trait of interest.
c. Example Dataset is Example_SNPs.csv

## Key Resources Table

**Table:** 

REAGENT or RESOURCE	SOURCE	IDENTIFIER
**Deposited Data**		

Example Datasets and code	https://github.com/blumsteinm/Projecting_MAF_ClimateChange	n/a

**Software and Algorithms**		

R v.3.6.0	R Core Team (2019). R: A language and environment for statistical computing. R Foundation for Statistical Computing, Vienna, Austria. URL https://www.R-project.org/	n/a
BiocManager v.1.30.10	Martin Morgan (2019). BiocManager: Access the Bioconductor Project Package Repository. R package version 1.30.10. https://CRAN.R-project.org/package=BiocManager	n/a
snpStats v.1.34.0	David Clayton (2019). snpStats: SnpMatrix and XSnpMatrix classes and methods. R package version 1.34.0.	n/a
data.table v.1.12.8	Matt Dowle and Arun Srinivasan (2019). data.table: Extension of “data.frame”. R package version 1.12.8. https://CRAN.R-project.org/package=data.table	n/a
vegan v.2.5-6	Jari Oksanen, F. Guillaume Blanchet, Michael Friendly, Roeland Kindt, Pierre Legendre, Dan McGlinn, Peter R. Minchin, R. B. O'Hara, Gavin L. Simpson, Peter Solymos, M. Henry H. Stevens, Eduard Szoecs and Helene Wagner (2019). vegan: Community Ecology Package. R package version 2.5-6. https://CRAN.R-project.org/package=vegan	n/a

## Step-By-Step Method Details

***Note:*** all steps are performed in the statistical computing environment “R”. Example code for each step can be found at https://github.com/blumsteinm/Projecting_MAF_ClimateChange/STAR_Protocol_Example_Code.R

### Calculate Minor Allele Frequencies by Population for Loci of Interest

**Timing: 10–30 min depending on file sizes**

This step uses R to pull information on the loci of interest from the genomic data files and uses it to calculate the minor allele frequency (MAF) by population.

1. Pull the allele information from the .bed files for each loci of interest using the “read.plink” function from the package *snpStats* in R.
a. If the allele information is in a large file, we recommend using “fread” from *data.table* instead of “read.csv” for faster loading.
b. Instead of reading the whole .bed file into R, use the “select.snps” parameter within “read.plink” to feed a list of loci of interest names or locations.
2. Calculate the MAF by population.
a. Merge the sample allele information with your population information so that you have a dataframe indicating the sample name, sample population, and what the samples’ alleles at each loci are.
i. Our species, *Populus trichocarpa*, is diploid. Thus our .bed files indicate any individual is either homozygous with 01 (AA) or 03 (BB) as the alleles value, or heterozygous with 02 (AB) as the alleles value. In our data, the minor allele is always 1/A.
b. Create a function for calculating the minor allele frequency by population.
i. freq <- function(alleles = NULL){
converted_alleles <- sapply(as.numeric( alleles ), function(x) ifelse(x == 1, 1, ifelse(x == 2, 0.5, ifelse(x == 3, 0, NA))))
allele_frequency <- sum(converted_alleles)/length(converted_alleles)
return(allele_frequency)
}
c. Use “aggregate” from base R and the “freq” function from above to calculate the MAF (allele A) by population

### Define the Major Axes of Climate Variation Using a Principal Component Analysis

**Timing: 10–20 min**

This step takes the climatic data from the past Normals data and future GCM projections and puts them into principal components (PC) space. PCs pull out major axes of explanatory variation, which is particularly useful when many climate variables are highly correlated and thus difficult to use in a statistical model.

**Figure 1:**
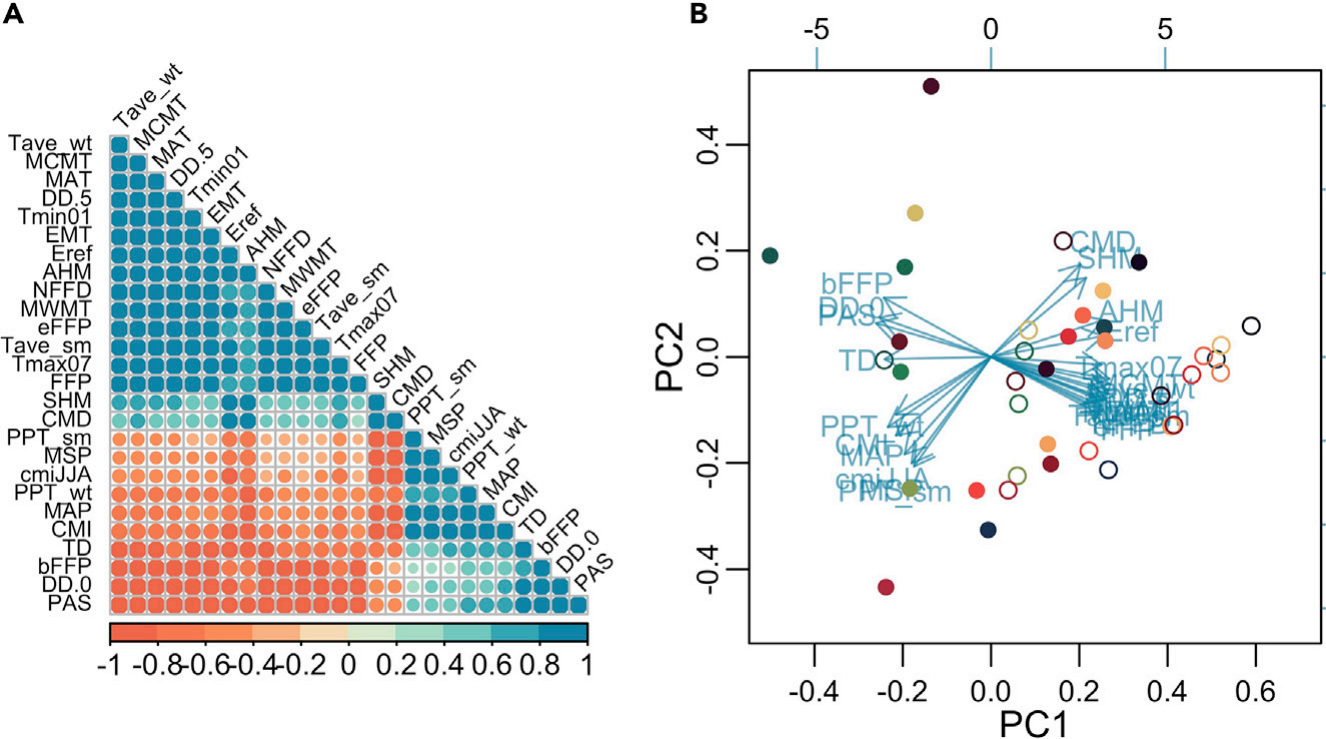
Visualizing Current and Future Climate in Principle Components Space A visualization of (A) climate variable correlations and (B) climate principal components space. The (A) correlations plot shows positive (blue) and negative (red) correlations between climate variables, with circle size and depth of color indicating the strength of the correlation. The (B) PCA plot depicts climate variables (blue arrows) in the first two axes of pc space. Where populations fall in PC space are shown with the colored dots, with the closed dots representing the past 30-years of climate data and their open counterparts representing where populations are expected to fall in the PC space in 2080.

3. Order rows in both climate files by population to ensure they match. This is essential for the principal component analysis (PCA) predictions.
4. Use “prcomp” from the *vegan* package to run a PCA of the past climate Normals data, inputting a matrix of all climate variables as the object.
a. Ensure that both “scale” and “center” are equal to TRUE. If not, climate variables with large values will disproportionately drive axis variation.
5. Use “predict” and the future climate data as “newdata” to project the future climate variables into PC space.
6. Check results using “biplot” from the *vegan* package (Figure 1).

### Fit a Canonical Correspondence Analysis to Current Allele Frequencies versus Climate

**Timing: 15–30 min**

This step uses the climate PCs and the MAFs by population that we have tabulated to fit a canonical correspondence analysis (CCA) model. This model is the relationship between past climate and current MAFs. This CCA will be used to project MAFs into the future under climate change.

7. Merge the past climate dataframe and MAFs dataframe using “merge” in base R. We merged based on “population name”, so that when data is put into models in subsequent steps, all columns are in the same order by population.
a. Record the index of the climate and MAF columns so that you can distinguish between the two when running the CCA.
8. Run a Null Model CCA with no predictors using “cca” from package *vegan.*
a. This will be used for model comparisons in a future step. In this case, no explanatory predictors are put into the model, just allele data.
b. We ran ours with 4,000 permutations.
9. Run a CCA with all climate predictors included using “cca” from package *vegan.*
a. Again, we ran this with 4,000 permutations.
10. Drop environmental predictors that are collinear/non-significant via a step-wise model comparison using “ordistep” in the *vegan* package.
a. We inputted the null CCA as the object, with the scope set to the full CCA predictor model.
b. We ran ours in both directions, forward and backward, then used the optimal model returned as our CCA model. Using “$anova” with the model object will return the significance of variables remaining in the model.

### Further Assess CCA Model Fit and Accuracy

**Timing: 20 min**

This step delves further into how much variation the CCA model explains and how well it does recapitulating our current data.

11. Calculate the percent of variation in the MAF data that the model explains as the constrained inertial value divided by the total inertia value.

***Note:*** The “total inertia” is the total variance in allele frequency distributions. The “constrained inertia” is the variance explained by the environmental variables.

**Figure 2:**
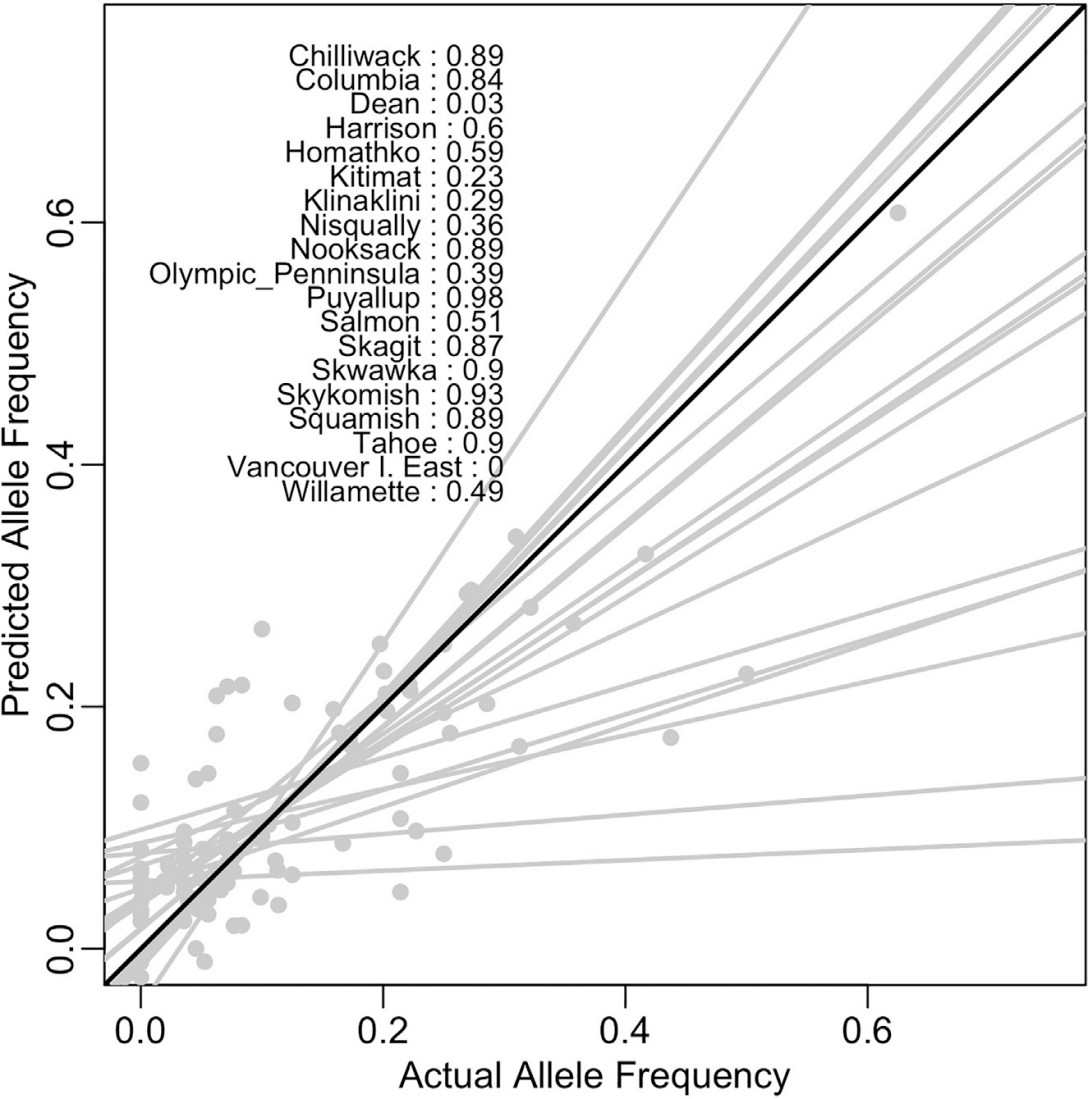
Predicted Minor Allele Frequencies under Past Climate Normals Plotted by Actual Current Minor Allele Frequencies Text shows the R^2^ of each population’s linear regression results. The black line indicates a 1:1 line, while gray lines are population-level fits.

12. Test whether the model explains more variation in MAF than expected by chance (p = 0.05) using “anova.cca” from the *vegan* package. If model is not significant, you should reevaluate the climate predictors that you are including.
13. Finally, visually compare to what degree predicted MAF values match actual MAF values, given the past climate Normals data (Figure 2).

a. We performed linear regressions by population to assess which populations were more poorly predicted than others.
b. In our example data, there are a few populations with non-significant p-values and low R^2^’s because we do not use the full dataset. These populations may need to be dropped in a real analysis should you find similar results in your data as the model is to replicating the pattern better than chance.

### Project Future Minor Allele Frequencies Given Predicted Climate Change

**Timing: 10 min**

This step uses the projected climate PCs and the CCA model formed in step 3 to predict MAF change in 60 years and calculate summary statistics.

**Figure 3:**
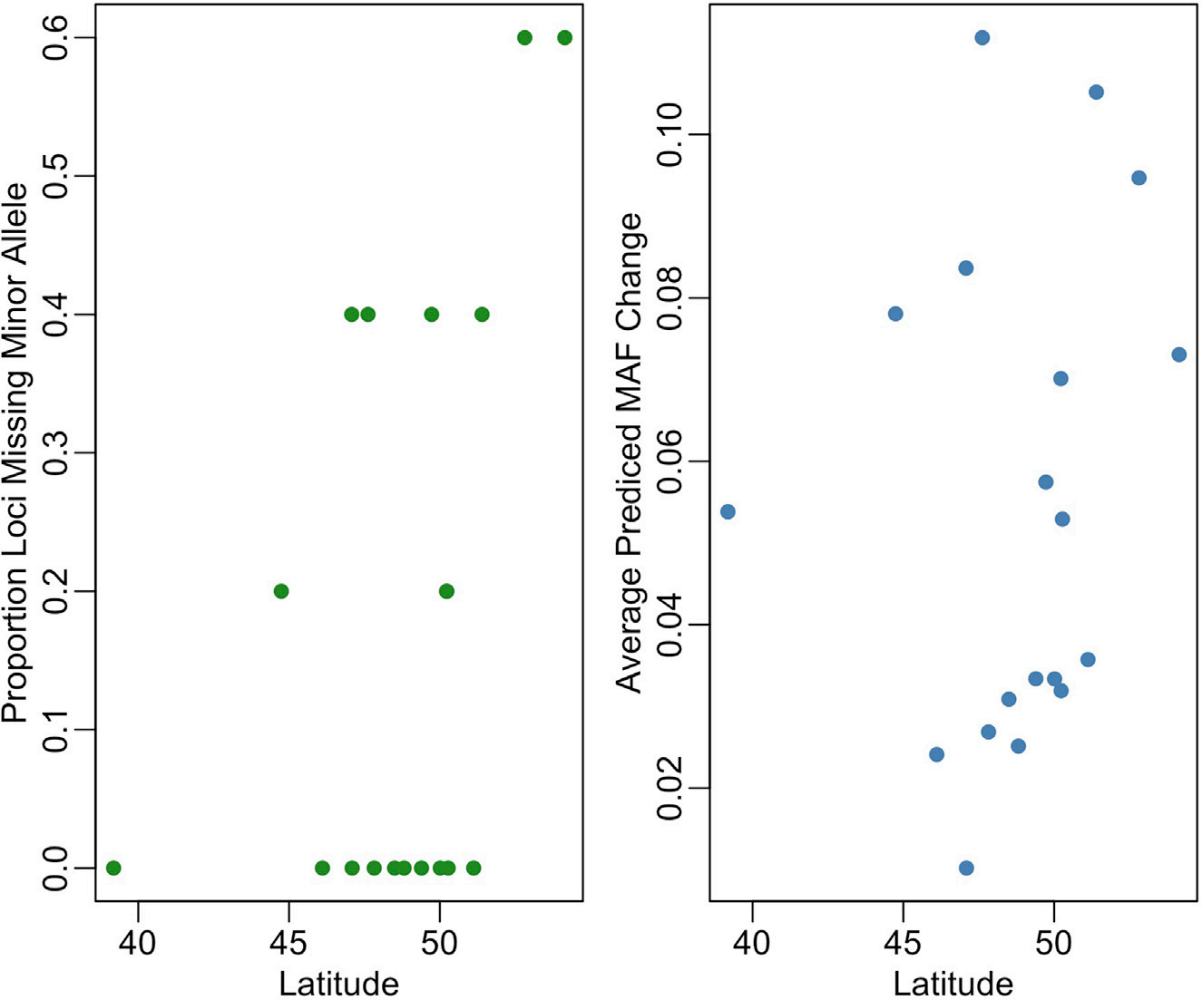
The Proportion of Loci Missing the Minor Allele and the Average Predicted MAF Change by Latitude Plots of (A) the proportion of loci that have only one allele by population and (B) the average project MAF change by population. Both are plotted against population latitude.

14. Use the “predict” function with CCA model created in step 3 and the future climate PC dataframe in the “newdata” parameter to predict MAF under future climate conditions.
15. Calculate the average predicted MAF change across all loci between the climate of the past 30 years and projected climate in 2080 (Figure 3).

a. Start by subtracting current MAFs from projected MAFs. This should result in a matrix of differences for each population (rows) at each loci (columns).
b. Sum the absolute value of each row to get average change by population.

***Note:*** we chose to look at the absolute value of change as we were interested in capturing both large increases *and* decreases in allele frequency.

16. Calculate the number of loci currently missing the minor allele by population (Figure 3).
a. We did this by setting all values in the current MAF matrix below 0.01 to 0 and all else to 1, then summing by populations (rows).

## Expected Outcomes

At the end of the process, you will have two dataframes; (1) the proportion of loci in each population that do not have the minor allele present and (2) average absolute change predicted in MAF between current and future climate (Data plotted by latitude in Figure 3). You will also generate a principal components model of past and projected climate (Figure 1 B) and a model of how Minor Allele Frequencies are associated with climate (Figure 2).

## Limitations

### Data Requirements

The greatest limitation of this approach are the substantial data requirements. A set of locally adapted loci are a starting requirement but are not easy to come by. For well documented model species, like *Arabidopsis thaliana*, there may be sets of genes or loci in the literature that have been experimentally validated and proven in trials to confer local adaptation in plants. For non-model organisms, QTL mapping or GWAS approaches must be taken to identify loci that are significantly associated with the adaptive phenotype and appear to be under selection (Q_st_ > F_st_). In this case, phenotypes must be measured on hundreds of genotypes grown in a common environment in order to pass significance thresholds (see our linked paper Blumstein et al., 2020).

In both model and non-model cases, extensive genomic information must be available for many individuals from different populations of the species, the number of which depends on the size of the genome. In the case of *Populus*, our GWAS set has 8.1 million SNPs, so we generally must sample at least 200–300 genotypes to have enough power to identify SNPs significantly associated with any given trait.

This method is ideally performed with full genome sequences, however reduced sampling representation data is also usable. In the latter case, it should be acknowledged that when missing portions of the genome, you may also be missing loci that are locally adapted. We have have provided some links below in “Resource Availability” to genomic information that may be useful in conducting such a project.

### Assumptions

This approach makes several key assumptions: 1) Individuals are diploid, ie. two allele options at any given loci. 2) MAFs are assumed to be distributed unimodally across environments (thus it would not be appropriate if the trait in question is under disruptive selection). 3) Relationships between MAF and environment are linear, no interactions between variables. 4) Allelic effects are independent and additive, no epistasis or dominance.

## Troubleshooting

If any of the model assumptions are violated, here are some alternative analysis options.

### Problem

MAFs are not unimodally distributed or relationships to environment are non-linear.

### Potential Solution

Utilize a different model other than a CCA that allows for non-linear relationships, such as a generalized dissimilarity models (GDM) or gradient forest (GF) as outlined in Fitzpatrick and Keller (2015).

### Problem

Loci interact epistatically.

### Potential Solution

Alter model to allow for multivariate responses of groups of genes (rather than individuals) to environment via an environmental co-association network analysis (Lotterhos et al., 2018).

### Problem

Loci of interest are not corrected for population structure.

### Potential Solution

If you do not account for population structure during your GWAS or QTL analyses, there are additional tools available to further refine your loci of interest and account for false positives. A number methods are available in R, such as the packages *LFMM* and *LEA* (both with the Bayesian bootstrap approach implemented), to account for the latent population structure of your data (Frichot and Francois, 2014, Caye and François, 2017).

## Resource Availability

### Lead Contact

Further information and requests for resources should be directed to and will be fulfilled by the Lead Contact, Meghan Blumstein, blumsteinm@gmail.com.

### Materials Availability

Here are some suggested places to get started looking for the various datasets you will need.

Climate data with past and project climate

North American Climate – Climate WNA (http://www.climatewna.com/)

Global – WorldClim (https://www.worldclim.org/data/index.html)

Genomic Resources (Reference Genomes and Mapping Populations)

JGI’s Phytozome (https://phytozome.jgi.doe.gov/pz/portal.html)

Hardwood Genomics (https://www.hardwoodgenomics.org/)

### Data and Code Availability

This protocol includes all example datasets and code needed to regenerate the figures and analyses outlined. The sample data and code are house at (https://github.com/blumsteinm/Projecting_MAF_ClimateChange).

## References

[bib1] Blumstein M., Richardson A.D., Weston D.J., Zhang J., Wellington M., Hopkins R. (2020). A new perspective on ecological prediction reveals limits to climate adaptation in a temperate tree species. Curr. Biol..

[bib2] Caye K., François O. (2017). http://membres-timc.imag.fr/Olivier.Francois/lfmm/index.htm.

[bib3] Fitzpatrick M.C., Keller S.R. (2015). Ecological genomics meets community-level modelling of biodiversity: mapping the genomic landscape of current and future environmental adaptation. Ecol. Lett..

[bib4] Frichot, E. & Francois, O. (2014). LEA: an R package for Landscape and Ecological Association studies. R package version 1.0*.* http://membres-timc.imag.fr/Olivier.Francois/lfmm/index.htm.

[bib5] Hamann A., Wang T., Spittlehouse D.L., Murdock T.Q. (2013). A comprehensive, high-resolution database of historical and projected climate surfaces for western North America. Bull. Am. Meteorol. Soc..

[bib6] Joint Genome Institute, D. O. E (2019). Phytozome 12 [Online]. https://phytozome.jgi.doe.gov/pz/portal.html.

[bib7] Lotterhos K.E., Yeaman S., Degner J., Aitken S., Hodgins K.A. (2018). Modularity of genes involved in local adaptation to climate despite physical linkage. Genome Biol..

[bib8] Wang T., Hamann A., Spittlehouse D.L., Murdock T.Q. (2012). ClimateWNA—high-resolution spatial climate data for Western North America. J. Appl. Meteorol. Climatol..

